# Prediction of age and sex from paranasal sinus images using a deep learning network

**DOI:** 10.1097/MD.0000000000024756

**Published:** 2021-02-19

**Authors:** Dong-Kyu Kim, Bum-Joo Cho, Myung-Je Lee, Ju Han Kim

**Affiliations:** a Department of Otorhinolaryngology-Head and Neck Surgery; b Division of Big Data and Artificial Intelligence, Chuncheon Sacred Heart Hospital; c Institute of New Frontier Research, Hallym University College of Medicine, Chuncheon; d Medical Artificial Intelligence Center, Hallym University Medical Center, Anyang; e Division of Biomedical Informatics, Seoul National University Biomedical Informatics (SNUBI), Seoul National University College of Medicine, Seoul, Republic of Korea.

**Keywords:** artificial Intelligence, deep learning, machine learning, neural networks, paranasal sinuses, sinus

## Abstract

This study was conducted to develop a convolutional neural network (CNN)-based model to predict the sex and age of patients by identifying unique unknown features from paranasal sinus (PNS) X-ray images.

We employed a retrospective study design and used anonymized patient imaging data. Two CNN models, adopting ResNet-152 and DenseNet-169 architectures, were trained to predict sex and age groups (20–39, 40–59, 60+ years). The area under the curve (AUC), algorithm accuracy, sensitivity, and specificity were assessed. Class-activation map (CAM) was used to detect deterministic areas. A total of 4160 PNS X-ray images were collected from 4160 patients. The PNS X-ray images of patients aged ≥20 years were retrieved from the picture archiving and communication database system of our institution. The classification performances in predicting the sex (male vs female) and 3 age groups (20–39, 40–59, 60+ years) for each established CNN model were evaluated.

For sex prediction, ResNet-152 performed slightly better (accuracy = 98.0%, sensitivity = 96.9%, specificity = 98.7%, and AUC = 0.939) than DenseNet-169. CAM indicated that maxillary sinuses (males) and ethmoid sinuses (females) were major factors in identifying sex. Meanwhile, for age prediction, the DenseNet-169 model was slightly more accurate in predicting age groups (77.6 ± 1.5% vs 76.3 ± 1.1%). CAM suggested that the maxillary sinus and the periodontal area were primary factors in identifying age groups.

Our deep learning model could predict sex and age based on PNS X-ray images. Therefore, it can assist in reducing the risk of patient misidentification in clinics.

## 1 Introduction

Deep learning involves computational models composed of multiple processing layers for learning representations of data with multiple levels of abstraction. This approach has dramatically improved object detection and recognition. Thus, multi-model deep learning architectures can contribute significantly toward the advancement of personalized medicine.^[1]^ Recently, among deep learning architectures, convolutional neural network (CNN) models have demonstrated superior performance compared to other machine-learning methods in object detection and recognition applications.^[2]^ Thus, these models are an effective solution for classification and recognition problems associated with large datasets. In addition, compared with other learning algorithms, the local receptive fields and shared weights of the CNN model are uniquely advantageous. Therefore, the CNN model is widely used for recognizing and differentiating medical images in clinical practice, including automated classification of gastric neoplasms based on endoscopic images, prediction of cardiovascular risk factors based on retinal fundus photographs, and classification of maxillary sinusitis based on paranasal sinus (PNS) X-ray images.^[3–5]^ Moreover, the CNN model features a majority decision area that uses a class activation map (CAM) from the tested dataset. It enables easy recognition of the area identified for the performance evaluation of feature prediction.

PNS X-ray is an imaging test that detects sinus problems such as sinusitis or mucosal thickening.^[6]^ PNS X-rays are less invasive and have relatively low amounts of radiation, as compared to other types of sinus tests. Additionally, in most cases, a PNS X-ray would be 1 test performed in a series of tests. For these reasons, it is frequently performed on an outpatient basis or as part of the patients’ stay in a hospital. In this context, for detecting unknown unique features, we propose a deep learning model to predict the sex and age of the patient from PNS X-ray images by using CNN. Furthermore, the proposed model determines the most effective decision area with a reasonable consensus based on CAM data. Thus, we expect that the proposed deep learning algorithms will help gain new insights into personalized medicine for patients with sinus problems.

## 2 Materials and methods

### 2.1 Subjects

This study included consecutive PNS images of patients who were clinically suspected to have rhinosinusitis, obtained between 2015 and 2018. We excluded patients younger than 20 years because the sinuses are not completely developed before this age. This study was approved by the Institutional Review Board of Hallym University Chuncheon Sacred Hospital (No. 2019-02-015). The Board waived the requirement of informed consent because of the retrospective study design and the use of anonymized patient imaging data. All PNS X-ray images, in JPEG format, with a mean resolution of 1600 × 1900 pixels, were retrieved from the image archiving and communication database system of the institution. Personal information or annotations were removed from the image during the extraction process. The effect of each subject on the deep learning event was assessed by randomly selecting a single PNS image for each participant. Inappropriate and illegible images, such as blurred or unfocused images, were excluded. Ultimately, a total of 4,160 PNS images of 4,160 patients were used in this study.

### 2.2 Dataset splitting and preprocessing

The entire dataset was divided into 3 subsets—training, validation, and test datasets—by random sampling with a ratio of 8:1:1, respectively. These sub-datasets were mutually exclusive. The validation dataset was used to find the optimal point in the training process. Each image was labeled according to sex and age. Specifically, we classified into3 age group categories: 20–39, 40–59, and 60+ years, because the morphology of paranasal sinus could be changed regarding increasing age before the analyses, all images were normalized using min–max normalization. Data augmentation was performed using vertical and horizontal flipping to increase the dataset to 4 times its original size.

### 2.3 Training CNN models

To construct machine-learning models, transfer learning was used. For this purpose, we adopted 2 CNN models with the ResNet-152 and DenseNet-169 architectures pretrained on the ILSVRC dataset. ResNet-152 is a modification of the residual network using skip connections (https://arxiv.org/abs/1603.05027), whereas DenseNet-169 is a modification of a previous CNN architecture obtained by connecting each layer to every other layer in a feed-forward fashion (https://arxiv.org/abs/1608.06993). Training was performed using a cyclical learning rate schedule.^[7,8]^ Four cycles were applied using cosine annealing with stochastic gradient restarts. Different learning rates were utilized for the low, middle, and high layers. In each cycle, early termination was initiated when the validation loss was minimized. For the training parameters, a dropout rate of 0.5, initial learning rate of 1e-3, and batch size of 6 were used. All the training was conducted on a PyTorch platform using a hardware system comprising an NVIDIA GeForce RTX 2080ti graphics processing unit and dual Xeon central processing units.

### 2.4 Performance evaluation and statistical analysis

The main outcome measurement was the classification performance for predicting the sex (male vs female) or 3 age groups (20–39, 40–59, 60+ years) for each established CNN model. After training the CNN models, the performance of each model was evaluated using the test dataset. To determine transformation-related uncertainty, we augmented the input image at the time of testing and obtained an estimation of the distribution of the prediction based on test-time augmentation.

The performance was evaluated by estimating the area under the curve (AUC). Furthermore, the sensitivity, specificity, positive predictive value (PPV), negative predictive value (NPV), and accuracy were calculated. The evaluation metrics were expressed as means ± standard deviation or means with 95% confidence intervals (CIs). The De Long test was used to compare the AUC values. A *P* value <.05 was considered to be statistically significant, and all the tests were two-sided.

## 3 Results

A total of 4160 images were included in the training dataset, and 416 images were used as the test dataset. The data composition of the training and test datasets is presented in Table 1. Female patients accounted for 61.3% of the dataset. The proportions of the subjects aged 20–39, 40–59, and 60+ years were 30.0%, 37.3%, and 32.7%, respectively. When using Resnet-152 and DenseNet-169 for classifying 1 image in the test dataset, the mean elapsed time was 0.270 ± 0.016 and 0.267 ± 0.020 s for sex prediction and 0.278 ± 0.020 and 0.284 ± 0.034 s for age prediction, respectively.

**Table 1:** Data composition of enrolled paranasal sinus views in the datasets.

	Whole dataset		Training set		Test set	
	Image N	Patient N	Image N	Patient N	Image N	Patient N
Overall	4160 (100.0%)	4160	3,744	3744	416	416
Male	1611 (38.7%)	1611	1,450	1450	161	161
Female	2549 (61.3%)	2549	2,294	2294	255	255
20–39	1248 (30.0%)	1248	1,123	1123	125	125
40–59	1550 (37.3%)	1550	1,395	1395	155	155
60+	1362 (32.7%)	1362	1,226	1226	136	136

### 3.1 Performance evaluation for sex prediction

**Figure 1 Heatmap of the confusion matrix for the per-category sensitivity of the ResNet-152 model for sex prediction:**
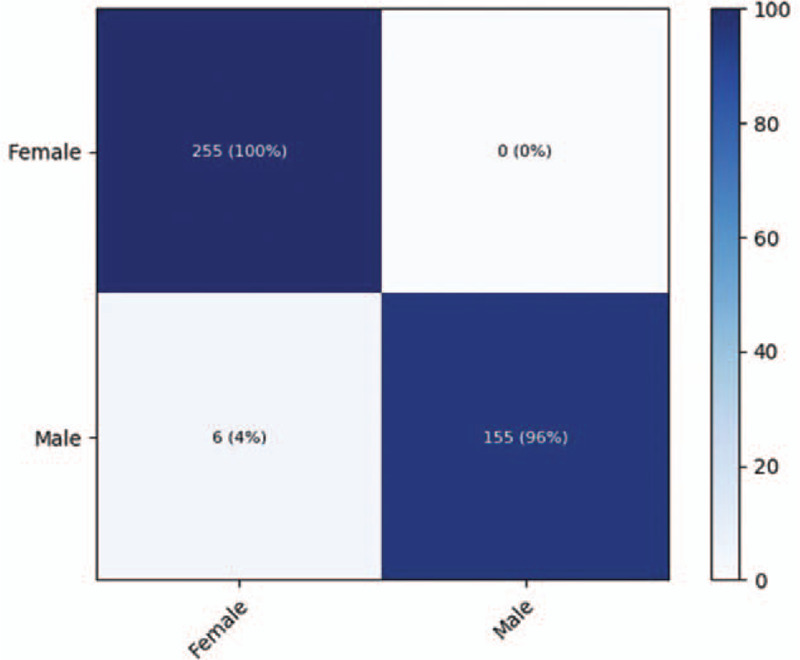
.

**Figure 2:**
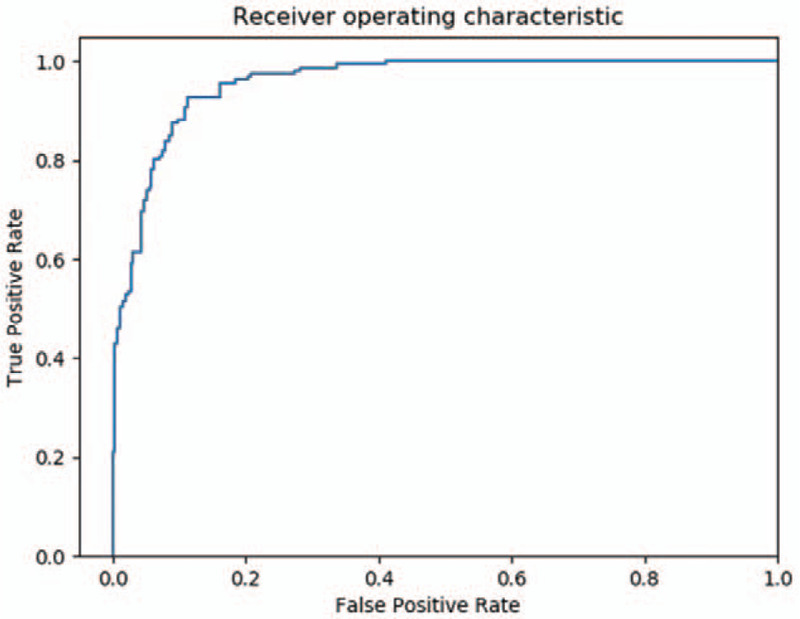
Receiver operating characteristic curves for the binary classification of sex (area under curves = 0.958).

**Figure 3:**
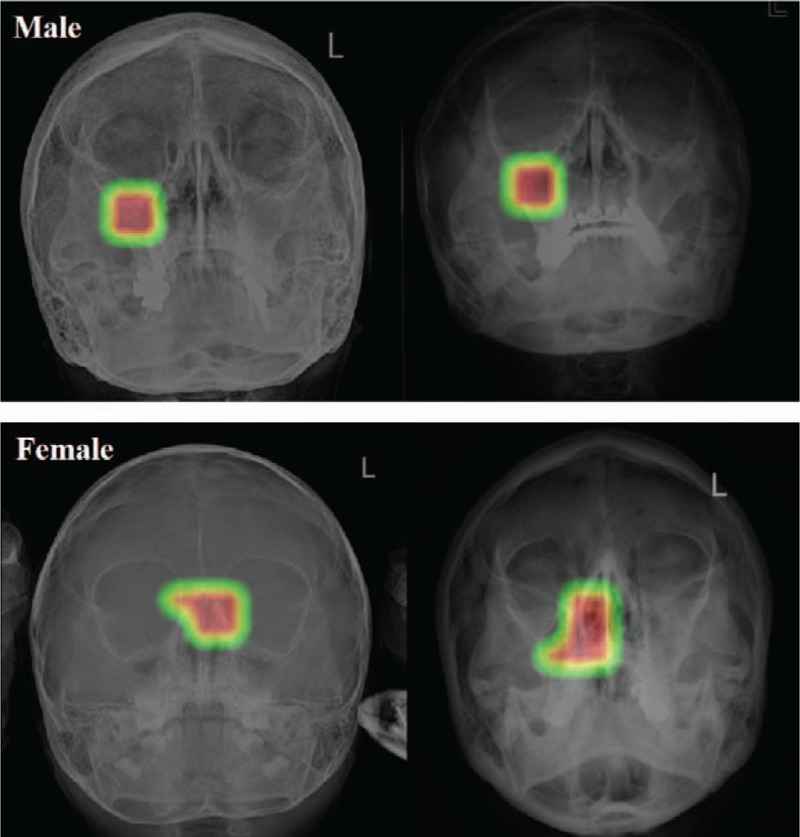
Majority decision areas for recognizing sex: class activation map in the convolutional neural network algorithm.

The performance of deep learning for sex prediction is summarized in Table 2. For the ResNet-152 model, the accuracy, sensitivity, and specificity for classifying a patient's sex were 98.0%, 96.9%, and 98.7%, respectively. The PPV and NPV were 97.9% and 98.1%, respectively. For the DenseNet-169 model, the accuracy, sensitivity, and specificity for sex classification were 97.7%, 97.5%, and 97.8%, respectively. The confusion matrix for the test dataset of the best-performing ResNet-152 model is presented in Figure 1. Additionally, the AUCs for sex classification were 0.939 (95% CI, 0.905–0.973) for the ResNet-152 model and 0.925 (95% CI, 0.891–0.959) for the DenseNet-169 model. The receiver operating characteristic (ROC) curve of the best performing ResNet-152 model for sex prediction is presented in Figure 2. Subsequently, to determine the discriminative image regions used by the CNN to identify that category, we performed a technique for generating CAM. Interestingly, we found that the maxillary sinuses for male prediction and the ethmoid sinuses for female prediction were activated on CAM (Fig. 3).

**Table 2:** Diagnostic performance for sex prediction of machine learning in each model (95% confidence interval).

Model	Accuracy (%)	Sensitivity (%)	Specificity (%)	PPV (%)	NPV (%)	AUC
Male vs. Female						
Resnet-152	98.0 (97.1–98.9)	96.9 (96.2–97.6)	98.7 (97.1–100)	97.9 (95.5–100)	98.1 (97.6–98.5)	0.939 (0.905–0.973)
DenseNet-169	97.7 (97.6–97.8)	97.5 (96.1–98.9)	97.8 (96.7–98.9)	96.5 (94.9–98.2)	98.4 (97.6–99.3)	0.925 (0.891–0.959)

### 3.2 Performance evaluation for age category prediction

**Figure 4 Heatmap of the confusion matrix for the per-category sensitivity of the DenseNet-169 model for age prediction:**
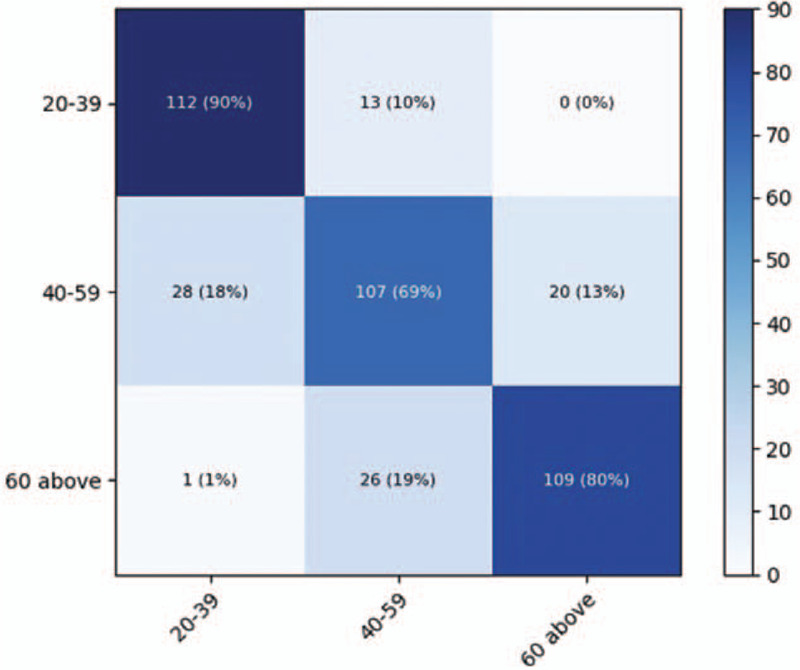
.

**Figure 5 Majority decision areas for recognizing age: class activation map in the convolutional neural network algorithm:**
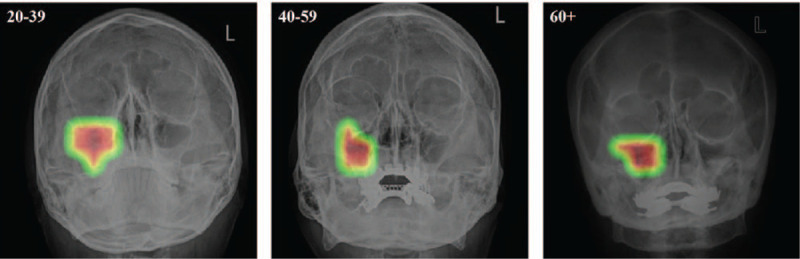
.

The performance of the DenseNet-169 model was higher, on average, than that of the Resnet-169 model, as presented in Table 3. The accuracy of the Resnet-152 and DenseNet-169 models for age prediction was 76.3 ± 1.1% and 77.6 ± 1.5%, respectively. For the young age group of 20 to 39 years, the accuracy, sensitivity, and specificity for age classification using the ResNet-152 model were 89.7%, 87.5%, and 90.7%, whereas the corresponding values were 89.7%, 88.0%, and 90.5%, respectively, for the DenseNet-169 model. The confusion matrix for the per-category sensitivity of the best-performing DenseNet-169 model in the test dataset is presented in Figure 4. Using the CAM technique, we observed that the majority of the discriminative image regions for classifying the age category were the maxillary sinus and periodontal areas on the test dataset (Fig. 5).

**Table 3:** Diagnostic performance for age prediction of machine learning in each model (95% confidence interval).

Model	Accuracy (%)	Sensitivity (%)	Specificity (%)	PPV (%)	NPV (%)	AUC
Resnet-152						
20–39	89.7 (89.3–90.1)	87.5 (85.6–89.4)	90.7 (89.2–92.1)	80.1 (77.8–82.3)	94.4 (93.7–95.2)	0.893 (0.887–0.899)
40–59	90.4 (89.7–91.0)	80.2 (77.2–83.2)	94.7 (94.2–95.3)	86.8 (85.4–87.8)	91.7 (90.5–92.9)	0.751 (0.750–0.752)
60+	86.8 (85.0–88.6)	81.2 (75.5–87.0)	89.5 (88.6–90.5)	79.3 (77.9–80.6)	90.7 (87.9–93.5)	0.859 (0.843–0.876)
DenseNet-169						
20–39	89.7 (88.9–90.6)	88.0 (84.9–91.1)	90.5 (90.0–90.9)	79.9 (79.2–80.6)	94.6 (93.3–95.9)	0.893 (0.878–0.908)
40–59	90.0 (89.2–90.8)	78.4 (77.1–79.6)	95.0 (94.0–96.0)	86.9 (83.5–90.3)	91.1 (90.5–91.7)	0.747 (0.722–0.772)
60+	87.5 (86.3–88.7)	84.1 (79.9–88.3)	89.2 (85.4–92.9)	79.3 (74.2–84.5)	92.1 (90.5–93.7)	0.866 (0.862–0.870)

## 4 Discussion

Deep learning is a machine-learning technique. It enables computational models composed of multiple processing layers to learn representations of data with multiple levels of abstraction.^[9]^ Thus, numerous researchers have begun focusing on deep learning as a promising technology to solve major problems in artificial intelligence. To apply deep learning systems for disease assessment using medical imaging, it is important to realize highly accurate classifications on test datasets as well as reasonable feature extraction of target lesions. However, traditional machine-learning methods for disease classification, such as support vector machines, K-means clustering, and the naïve Bayes classifier, require expert knowledge and time-consuming manual adjustments to extract specific features.^[10–12]^ This implies that traditional machine-learning methods require the extraction of features representing characteristics by using various segmentation methods. Thus, recent deep learning architectures can facilitate the direct acquisition of useful feature representations from data. Among these architectures, the CNN model is one of the more powerful imaging classifiers. Thus, it is widely used to analyze radiologic images, such as those obtained using X-ray, computerized tomography, and magnetic resonance imaging.^[13]^ In addition, CAM enables classification-trained CNNs to learn to localize visual objects without using any bounding box annotations.^[14]^ In the present study, we constructed a relatively large dataset comprising PNS X-ray images. The majority decision algorithm was shown to be the most efficient model for classifying these images in the specified sex and age categories.

To the best of our knowledge, the present study is the first to develop a deep learning model for the prediction of sex and age. Our evaluation of the accuracy of the models showed that the CNN-based classifier for sex achieved the highest AUC of 0.939 (95% CI 0.905–0.973) on the ResNet-152 model, whereas the highest accuracy obtained by the DenseNet-169 model was 77.6 ± 1.5% for classifying the age category. Interestingly, we found that the maxillary and ethmoid sinuses were used predominantly for classifying sex, whereas a majority of the decisions pertaining to age classification were based on the maxillary sinus and periodontal areas. These findings suggest that the CNN-based deep learning approach can effectively identify the sex and age categories based on PNS X-ray imaging features. We believe that our novel findings can assist in reducing the risk of patient misidentification. Patient identification errors have been one of the most serious healthcare quality issues for patient safety worldwide. Currently, despite the advances in technology or approaches used for accurately identifying patients, patient identification errors often occur due to increases in the workload of the medical staff. Thus, during the PNS X-ray test, our deep learning model could ensure accurate patient identification prior to any medical intervention and provide safer care with significantly fewer errors.

However, our studies still have several limitations. First, our deep learning model could identify only 3 age groups. Thus, as the amount of information that is learned increases, it is necessary to evolve the algorithm based on more detailed age groups. Second, our deep learning model could not differentiate sex on children group. In the future, developments that can identify sex on children's age are also needed. Finally, in the current state, this approach would not directly assist clinical treatments, although our deep learning model provides novel information for identifying anatomical regions. Thus, we have a plan for developing the algorithm that could make the predictions of clinical outcomes.

## 5 Conclusion

In the present study, our proposed CNN model showed excellent performance in predicting the sex and age categories. Additionally, we found that certain paranasal sinuses are major deterministic areas for the prediction of sex and age. Therefore, we expect that it will help reduce the risk of patient misidentification during PNS X-ray tests.

## Author contributions

**Conceptualization:** Dong-Kyu Kim, Bum-Joo Cho.

**Data curation:** Dong-Kyu Kim, Bum-Joo Cho.

**Formal analysis:** Dong-Kyu Kim, Bum-Joo Cho.

**Funding acquisition:** Dong-Kyu Kim, Bum-Joo Cho.

**Investigation:** Dong-Kyu Kim, Bum-Joo Cho.

**Methodology:** Bum-Joo Cho, Myung-Je Lee.

**Project administration:** Dong-Kyu Kim, Bum-Joo Cho.

**Resources:** Bum-Joo Cho.

**Software:** Myung-Je Lee.

**Supervision:** Dong-Kyu Kim, Ju Han Kim.

**Validation:** Bum-Joo Cho, Myung-Je Lee.

**Visualization:** Myung-Je Lee.

**Writing – original draft:** Dong-Kyu Kim, Bum-Joo Cho.

**Writing – review & editing:** Dong-Kyu Kim, Bum-Joo Cho.
